# *Mycoplasma pneumoniae* as a causative agent of community-acquired pneumonia in children: clinical features and laboratory diagnosis

**DOI:** 10.1186/s13052-014-0104-4

**Published:** 2014-12-18

**Authors:** Biljana Medjo, Marina Atanaskovic-Markovic, Snezana Radic, Dimitrije Nikolic, Marija Lukac, Slobodanka Djukic

**Affiliations:** Medical Faculty, University of Belgrade, Dr Subotica 8, 11 000 Belgrade, Serbia; University Children’s Hospital, Tirsova 10, Belgrade, Serbia; Children’s Hospital for Respiratory Diseases and TB, Clinical Health Center “Dr Dragisa Misovic - Dedinje”, Jovana Marinovica 4, 11 000 Belgrade, Serbia; Institute of Microbiology, Medical Faculty, University of Belgrade, Dr Subotica 8, 11 000 Belgrade, Serbia

**Keywords:** Children, Community-acquired pneumonia, Diagnosis, *Mycoplasma pneumoniae*, Polymerase chain reaction

## Abstract

**Background:**

*Mycoplasma pneumoniae* is a common cause of community-acquired pneumonia (CAP) in children. The aim of this study was to assess the prevalence of *Mycoplasma pneumoniae* infection in children with CAP and find clinical, radiological and laboratory features helpful to diagnose *Mycoplasma pneumoniae* pneumonia. Furthermore, we evaluated the value of serology, real-time PCR (RT-PCR) and culture for the accurate diagnosis of *Mycoplasma pneumoniae* pneumonia.

**Methods:**

The study included 166 children aged between 1 and 15 years with radiologically confirmed pneumonia. Throat swab specimens were cultured and assessed by RT-PCR for the presence of *Mycoplasma pneumoniae. Mycoplasma pneumoniae*-specific IgM and IgG antibodies were determined using ELISA in paired sera.

**Results:**

*Mycoplasma pneumoniae* pneumonia was diagnosed in 14.5% CAP cases. Cough (p=0.029), headache (p=0.001) and wheezing (p=0.036) were more frequent in children with *Mycoplasma pneumoniae* pneumonia compared to children with pneumonia caused by other pathogens. Logistic regression analysis showed that headache (odds ratio [OR] =36.077, p=0.001) and wheezing (OR=5.681, p=0.003) were significantly associated with MP pneumonia. Neither radiological findings, nor common laboratory parameters distinguished *Mycoplasma pneumoniae* infection in children with CAP. Using IgG serology in paired sera as the gold standard, we found that sensitivity of IgM serology, RT-PCR and culture was equal (81.82%), while specificity values were 100%, 98.6% and 100% respectively. We observed that combination of IgM detection in acute-phase serum and RT-PCR was positive for 91.7% of cases with *Mycoplasma pneumoniae* infection.

**Conclusions:**

There are no characteristic radiological findings, or routine laboratory tests that would distinguish CAP caused by *Mycoplasma pneumoniae* from other CAP. It was found that clinical features such as headache and wheezing are indicative for *Mycoplasma pneumoniae* infection. Furthermore, it was found that during the acute phase of disease, detection of IgM antibodies in combination with RT-PCR allows for precise and reliable diagnosis of *Mycoplasma pneumoniae* infections in children.

## Background

*Mycoplasma pneumoniae* (MP) causes up to 40% of community-acquired pneumonia (CAP) in children and about 18% of infections in patients requiring hospitalization [1]. In the majority of cases of suspected MP pneumonia, the presumptive diagnosis is made on clinical and radiological findings alone. However, MP is not susceptible to the β-lactam antibiotics regularly used for the treatment of pediatric pneumonia [2], therefore a specific diagnostic tool is extremely important. Laboratory diagnosis of MP infection in clinical practice have been based on serology, culture and polymerase chain reaction (PCR), although each of them has specific limitations [3].

In Serbia, however, diagnosis of MP infection is most often based on serology. Consequently, the precise incidence of MP infection in children with CAP remains unknown. Therefore, we conducted a study to assess the incidence of MP pneumonia, define clinical, radiological and laboratory features specific for MP pneumonia and to evaluate the value of serology, real-time PCR (RT-PCR) and culture for the accurate diagnosis of MP pneumonia.

## Methods

### Study population

Previously healthy children aged between 1 and 15 years with symptoms, signs and chest radiographs consistent with community-acquired pneumonia examined in the Emergency department of University Children’s Hospital in Belgrade from April 2012 to March 2014 were prospectively enrolled in this study. Pneumonia was defined as the presence of fever, acute respiratory symptoms (cough, tachypnoea, difficult breathing) or both, plus presence of new infiltrate on chest radiography or consolidation not attributable to some other aetiology [4]. Children who received anti-mycoplasma therapy were excluded from the study. This study was approved by Ethics Committee of University Children’s Hospital in Belgrade. Patients were enrolled in study after written informed consent had been obtained from their parents or guardians.

Demographic and clinical data were collected uniformly from all children who were included in the study. Chest radiographs were taken and reviewed by pediatric radiologist. Radiological findings were classified as interstitial infiltration, linear opacities, patchy infiltration, segmental or lobar consolidation, reticulo-nodular infiltrate or pleural effusion [5]. Throat swabs taken at enrolment were cultured and assessed by RT-PCR. Blood samples were obtained at the time of enrolment and two to four weeks after. White blood cell (WBC) count, C-reactive protein (CRP) and serological tests were performed at enrolment. The patients were hospitalized on the basis of the clinical decision of the pediatricians on duty. Data regarding clinical course of illness were also recorded.

### Culture methods

Clinical specimens were inoculated on PPLO (Pleuro pneumonia like organisms) broth and agar bases (HiMedia, HiMedia Laboratories, Mumbai, India) supplemented with *Mycoplasma* supplements (HiMedia) and glucose (0.1%) (HiMedia). The cultures were incubated at 37°C in an atmosphere of 5% CO_2_ and 95% air. The broth cultures were observed daily for 21 days and the growth of MP is indicated by phenol red (acid production because of glucose degradation leads to a yellowing of the broth). The agar cultures were examined microscopically after 21 days of incubation for the presence of typical colonies (the isolated colonies were identified by their characteristic homogeneous granular appearance and stained with Diene’s staining).

### DNA extraction

The Qiagen QIAamp DNA minikit (QIAGEN, Hilden, Germany) was used for nucleic acid extraction from examined respiratory specimens according to the manufacturer’s instructions. The elution volume was reduced to 30 μl.

### Real-time PCR

Each nucleic acid extract from examined respiratory specimens was tested with our in-house real-time PCR assay. This method, targeting a 125-bp fragment of the *M. pneumoniae* P1 cytadhesin gene, was performed using the primers P1-204 [5′-GTGAACGTATCGTAACACGAGCTTT-3′), P1-328 (5′-TGGTTTGTTGACTGCCACTGC-3′], the TaqMan probe P1-284R [5′-TAM-TTGTCGCGCACTAAGGCCCACG-MGB-3′], and a commercial internal control (IC), the TaqMan exogenous internal positive control (VIC dye) (Applied Biosystems, Foster City, USA). Real-time PCR was performed in 25 μl of a reaction mixture consisting of 12.5 μl of 2X TaqMan universal PCR master mix (Applied Biosystems, Foster City, USA), 0.9 μM of each primer, 0.25 μM of probe, 0.5 μl of IC DNA, 2.5 μl of IC primers/probe, and 5 μl of extracted DNA. Amplifications were performed using a Applied Biosystems 7500 Real-Time PCR system (Applied Biosystems, Foster City, USA) under the following conditions: 2 min at 50°C and 10 min at 95°C, followed by 45 cycles of 15 s at 95°C and 1 min at 60°C.

### Serological testing

Serum was separated from the venous blood samples and stored at -20C° till assayed. For detection of MP antibodies, a commercially available IgM and an IgG enzyme-linked immunosorbent assay (ELISA Euroimmun, Lübeck, Germany) were performed. The serological diagnosis was defined when IgM antibodies were detected (>1.1) in single serum or if at least 4-fold increase in the IgG antibody titer was detected in the paired sera.

### Definition of acute MP infection

Acute MP infection was defined if at least one method was positive among detection of MP by culture or by PCR or detection of MP-specific antibodies by serology.

### Statistical analysis

Clinical data of the separate groups were described my mean values and standard deviations or percentages, according to the type of variable. Student’s *t*-test and ManneWhitney *U*-test were used to compare continuous variables between the group and Chi-square test and Fisher’s exact test were used for categorical variables. Furthermore, the variables resulting significant at the univariate analysis were subjected to logistic regression and odds ratios and their 95% confidence interval (CI) were calculated. A p value of <0.05 was considered statistically significant. Sensitivity and specificity were calculated using SS calculation comparing truly/falsely positive/negative with frequencies of referent methods (golden standard).

## Results

Current study included 166 children with CAP. Among them 86 (51.8%) were males and 80 (48.2%) were females (p=0.641). The mean age of the study population was 6.35 ± 4.52 years, 88 (53.0%) children were less than 5 years old, while 78 (47.0%) were 5–15 years old (p=0.438). *Mycoplasma pneumoniae* pneumonia was diagnosed in 24 (14.5%) children according to our diagnostic criteria, with significant difference in the age distribution, 25% of them were below 5 years and 75% were 5 years and over (p=0.003). *Mycoplasma pneumoniae* infections were detected throughout the year. A highest proportion of MP infections was recorded in the autumn (33.3%), followed by 29.2% in winter, 25% in spring and 12.5% in summer but the difference was not statistically significant (p=0.506).

In our study MP infection was present in boys more often than in girls (p=0.014). Children with MP pneumonia were significantly older (p=0.001), and had longer duration of fever (p=0.021) and cough before enrolment (p=0.026) compared to children with non-MP pneumonia (Table 1).

**Table 1:** **Comparison of the demographic and clinical data between children with** ***Mycoplasma pneumoniae*** **pneumonia and in children with pneumonia caused by other pathogens at enrolment**

	***Mycoplasma pneumoniae*** **pneumonia**	**Non-** ***Mycoplasma pneumoniae*** **pneumonia**	**P value**
	**(n=24)**	**(n=142)**	
Sex (%)			
Males	75	47.9	p=0.014
Females	25	52.1	
Age (years)	9.89 ± 5.21	5.75 ± 4.13	p=0.001
Duration of symptoms before enrolment (days)	7.33 ± 4.89	5.75 ± 3.78	p=0.089
Fever duration before enrolment (days)	5.50 ± 3.65	3.83 ± 2.93	p=0.021
Cough duration before enrolment (days)	7.17 ± 4.89	5.01 ± 4.25	p=0.026

Date are presented as mean ± SD unless otherwise indicated.

Clinical characteristics of children in both groups were further analyzed and compared. We found that cough (p=0.029), headache (p=0.001) and wheezing (p=0.036) were more frequent in children with MP pneumonia compared to children with non-MP pneumonia (Table 2).

**Table 2:** **Comparison of the symptoms, signs and physical findings between children with** ***Mycoplasma pneumoniae*** **pneumonia and in children with pneumonia caused by other pathogens at inclusion**

	***Mycoplasma pneumoniae*** **pneumonia**	**Non -** ***Mycoplasma pneumoniae*** **pneumonia**	**P value**
	**(n=24)**	**(n=142)**	
Fever	20 (83.3)	132 (93.0)	p=0.117
Cough	24 (100.0)	118 (83.1)	p=0.029
Coryza	4 (16.7)	36 (25.3)	p=0.357
Difficulty breathing	2 (8.3)	16 (11.3)	p=0.669
Headache	6 (25.0)	2 (1.4)	p=0.001
Vomiting	4 (16.7)	30 (21.1)	p=0.617
Tachypnoea	4 (16.7)	32 (22.5)	p=0.649
Rales	8 (33.3)	40 (28.1)	p=0.606
Wheezing	8 (33.3)	22 (15.5)	p=0.036
Atopy or asthma	12 (50.0)	60 (42.3)	p=0.479

Date are presented as number (%).

We did not observe other significant differences regarding symptoms, signs or physical findings between these two groups (Table 2). The variables found to be more frequent in children with MP pneumonia were further analyzed by logistic regression. It was found that headache (odds ratio [OR] =36.077, 95% confidence interval [CI] =4.897-265.811, p=0.001), wheezing (OR=5.681, 95% CI=1.776-18.175, p=0.003), mail gender (OR=0.162, 95% CI=0.048-0.548, p=0.003) and age 5 years and older (OR=3.067, 95% CI=1.016-9.251, p =0.047) were significantly associated with MP pneumonia.

The radiological findings in children with MP pneumonia were variable and summarized in Table 3. No significant difference was observed in relation to the radiological findings in the children with MP pneumonia and those with pneumonia caused by other pathogens. We noticed that unilateral lung involvements were more often than bilateral in children with and without MP infection (83.3% vs. 16.7%, 70.4% vs. 29.59%, p=0.191 respectively). Furthermore, 58.3% children with MP pneumonia had radiological findings on the right side compared to 47.9% children with non-MP infection although the difference did not reach statistical significance (p=0.418). We did not find a difference in total and differential WBC, platelet and CRP levels between study groups (Table 3). There were no significant associations between radiological or laboratory findings and MP infection as well.

**Table 3:** **Comparison of the radiological characteristics and laboratory data between children with** ***Mycoplasma pneumoniae*** **pneumonia and in children with pneumonia caused by other pathogens**

	***Mycoplasma pneumoniae*** **pneumonia**	**Non -** ***Mycoplasma pneumoniae*** **pneumonia**
	**(n=24)**	**(n=142)**
Radiological characteristics		
Linear opacities	12 (50.0)	83 (58.4)
Patchy infiltration	4 (16.7)	18 (12.7)
Segmental or lobar consolidation	2 (8.3)	22 (15.5)
Interstitial infiltration	2 (8.3)	11 (7.7)
Reticulo-nodular infiltrate	2 (8.3)	6 (4.2)
Pleural effusion	2 (8.3)	2 (1.4)
WBC (10^9^/L, mean ± SD)	13.20 ± 8.28	16.81 ± 10.51
Neutrophils (%)	69.37 ± 19.35	68.96 ± 18.60
Lymphocytis (%)	24.03 ± 17.25	24.88 ± 15.66
Monocytes (%)	5.82 ± 3.50	4.65 ± 2.73
Platelet (10^9^/L, mean ± SD)	280.00 ± 109.37	273.83 ± 98.52
CRP (mg/L, mean ± SD)	51.75 ± 68.29	90.97 ± 96.92

Date are presented as number (%) unless otherwise indicated.

WBC: white blood cell count; CRP: C-reactive protein; * p < 0.05.

Moreover, we analyzed group of children less than 5 years old and found that the proportion of MP pneumonia in this age group was 6.8%. Interestingly, in children 5 years and older, 23.1% cases of CAP were caused by MP. Additionally, we compared clinical characteristics of children with MP pneumonia in these two groups and found that 66.7% of children below 5 years had coryza and 33.3% had difficulty breathing, while none of the older children had those symptoms (p=0.001, p=0.011, respectively). The younger children were more likely than the older children to have tachypnoea (33.3% vs. 11.1%: p=0.031) as well. There were no other differences regarding clinical characteristics, radiological or laboratory findings when these two groups of children were compared.

Hospital admission between children with and without MP pneumonia showed no differences (91.7% vs. 88.7%, p=0.669). However, children with MP infection had longer total duration of fever and cough compared to children with non-MP infection (6.33 ± 3.73 vs. 4.68 ± 3.23, p=0.02; 16.04 ± 5.38 vs. 8.32 ± 5.51, p=0.015 respectively) Moreover children with MP pneumonia had longer duration of wheezing and length of hospitalization compared to children with non-MP pneumonia (2.25 ± 3.33 vs. 0.59 ± 1.42, p=0.001; 7.33 ± 3.40 vs. 3.48 ± 2.78, p=0.001 respectively). None of children with MP pneumonia exhibited extrapulmonary manifestations. All children recovered without any of complication associated with MP infection.

**Figure 1:**
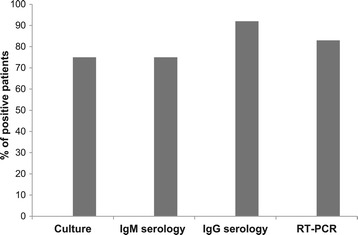
**In a group of patients with** ***Mycoplasma pneumoniae*** **pneumonia, percents of positive patients for each method.** RT-PCR: real-time polymerase chain reaction

Out of 24 children with MP pneumonia, 18 (75.0%) were confirmed to be MP positive by serology, RT-PCR and culture, 4 (16.7%) children were serologically positive only and 2 (8.3%) were positive by RT-PCR only (Figure 1). Real-time PCR was positive in 81.8% serologically positive children. In all 20 RT-PCR positive children, the RT-PCR was performed 7.8 ± 5.2 days after disease onset. A specific IgM antibody response in acute-phase sera was detected in 18 of 22 (81.8%) serologically positive children whose acute-phase sera were taken 8.7 ± 4.8 days after disease onset. All 22 serologically positive children had fourfold increase in IgG antibody titers in convalescent-phase sera. The mean value of IgM and IgG were 1.65 ± 1.16 and 87.58 ± 40.77 respectively. Moreover, we compared diagnostic value of three different methods, IgM serology in acute-phase sera, RT-PCR and culture, considering a fourfold or greater increase of IgG antibody titers in paired sera as the gold standard (Table 4). As shown in Table 5 sensitivity and specificity of all three methods were similar. On the contrary, when RT-PCR was considered as the gold standard IgM serology had the lowest sensitivity (Table 6). Additionally, we observed that combination of IgM detection in acute-phase serum and RT-PCR revealed positive results for 22 (91.7%) of 24 cases with MP infection.

**Table 4:** ***Mycoplasma pneumoniae*** **detection by IgM antibody testing, real-time polymerase chain reaction, culture and IgG antibody titers in paired sera**

		**IgG antibody titers in paired sera with a fourfold increase,**	
		**Positive**	**Negative**
IgM resulte	Positive	18 (10.84)	0 (0)
	Negative	4 (2.41)	144 (86.75)
RT-PCR results	Positive	18 (10.84)	2 (1.2)
	Negative	4 (2.41)	142 (85.54)
Culture results	Positive	18 (10.84)	0 (0)
	Negative	4 (2.41)	144 (86.75)

Date are presented as number (%).

RT-PCR: real-time polymerase chain reaction.

**Table 5:** **Diagnostic values of different methods with a fourfold or greater increase of IgG antibody titers in paired sera as the gold standard**

	**Sensitivity (%)**	**Specificity (%)**	**PPV (%)**	**NPV (%)**	**PLR**
Serology (IgM antibodies)	81.82	100.00	100.00	97.30	
RT-PCR	81.82	98.61	90.00	97.26	58.91
Culture	81.82	100.00	100.00	97.30	

Date are presented as percentage.

RT-PCR: real-time polymerase chain reaction; PPV: positive predictive value; NPV: negative predictive value; PLR: positive likelihood ratio.

**Table 6:** **Diagnostic values of different methods with a real-time polymerase chain reaction as the gold standard**

	**Sensitivity (%)**	**Specificity (%)**	**PPV (%)**	**NPV (%)**	**PLR**
Serology (IgM antibodies)	80.00	98.63	88.89	97.30	58.40
Serology (fourfold increase IgG antibodies)	90.00	97.26	81.82	98.61	32.85
Culture	90.00	100.00	100.00	98.65	

Date are presented as percentage.

RT-PCR: real-time polymerase chain reaction; PPV: positive predictive value; NPV: negative predictive value; PLR: positive likelihood ratio.

## Discussion

In this study we assessed the incidence of MP pneumonia, analyzed clinical, radiological and laboratory characteristics of MP pneumonia and compared value of three different diagnostic methods.

We found that 14.5% cases of CAP in Serbia were caused by MP. Most hospital-based studies from Europe reported similar frequencies of MP pneumonia with rates ranging from 9% to 14% [6-11]. On the contrary, studies from Asia reported higher prevalence of MP pneumonia [12-15]. However, comparison with results obtained from other studies is difficult due to the heterogeneity of the epidemiologic conditions, studied population, diversity in clinical specimens, and diagnostic methods applied. Among MP positive children in present study, 75% were children between 5–15 years old. In accordance to our data various studies reported that children 5 years and older have a higher rate of MP infections compared to younger children [6,9,10,16]. We observed that there was no seasonal variation in MP infection which was also found in studies performed in Italy and Tunis [17,18].

The clinical presentation of MP pneumonia observed in our study did not differ from those described in other studies [9,10,14,19]. One third of children with MP pneumonia had wheezing whereas fever and cough were the most common symptoms. When we compared clinical characteristics between children with MP pneumonia and non-MP pneumonia, we found that cough, headache and wheezing were more frequent in children with MP infection. Studies from Turkey and Brazil found that cough was contributing finding to MP infection [16,20].

Higher incidence of wheezing in children with MP pneumonia in our study could be due to the fact that half of children with MP infection had asthma or atopy. However, children with non-MP pneumonia had high incidence of asthma or atopy as well (42%) and no significant difference was found between this two groups. Moreover, logistic regression analysis showed that wheezing was significantly associated with MP pneumonia. Italian study found association between MP infection and wheezing in children 5 years old and over [21]. Furthermore, we observed that the radiological and laboratory findings did not distinguish MP pneumonia from other CAP as reported elsewhere [10,14,17]. Although the sample size of children less than 5 years old was small, we found that younger children more often had coryza, difficulty breathing and tachypnoea which is in agreement with previous reports [9,22]. The fact that all children below 5 years were hospitalized and probably had more severe disease could have contributed to this observation. Extrapulmonary manifestations were not observed in present study as previously reported [19].

Detection of IgM, in a single serum for diagnosis of MP infection is considered significant in children that had fewer opportunities for repeated exposure [23]. The sensitivity and specificity of IgM serology in present study were 81.82%, and 100% respectively compared to IgG serology and 80.00% and 98.63% respectively compared to RT-PCR. Nadal et al. reported sensitivity and specificity of IgM serology related to serology of 78.1% and 87.1% respectively in children with MP pneumonia [7]. Chang et al. used RT-PCR as a reference method and observed that sensitivity and specificity of IgM serology were 62.2% and 85.5% respectively [24]. Sensitivity of serology could be affected by timing of specimen collection. This could explain high sensitivity of IgM serology in present study, considering that sera were taken in the second week after disease onset. According to the literature detection of IgG antibodies in paired sera is more sensitive method, which was also confirmed by our results [25].

Our results show that sensitivity and specificity of RT-PCR compared to IgG serology were 81.82% and 98.61% respectively. Nadal et al. found similar sensitivity and specificity of RT-PCR compared to serology in children with MP pneumonia [7]. Morozumi et al. reported higher sensitivity and similar specificity of RT-PCR related to serology, 90.2% and 97.9% respectively [26]. However, some recent studies report lower sensitivity and specificity of this method compared to IgM serology [24,27] and compared to IgG serology [28,29]. This heterogeneity between studies regarding sensitivity of PCR method could be due to the different PCR types, different gene targets or different sample type and time point for sampling [30]. According to the literature RT-PCR revealed higher sensitivity compared to others types of PCR [30,31], but this could only partially explain our results. Finally, we found that combination of IgM detection in acute-phase serum and RT-PCR was positive for 91.7% cases in children with MP infection indicating a possible use of both techniques as a valid diagnostic approach in early detection of MP infection in children with CAP.

However, present study showed some discrepancies between the results of the diagnostic methods used. The 4 cases of negative RT-PCR results with positive serology may be due a disappearance, or a low level of MP load in the sample [25]. The 2 positive RT-PCR results in serologically negative children may be due to an inadequate immune response or detection of a carrier state [32].

Our study had some limitations. One of them is the small sample size. Besides, the results might be limited due to the fact that children in this study were presenting to a tertiary children hospital, therefore patients with more severe infections may have been overrepresented. Furthermore, we did not perform extensive microbiological testing, therefore we cannot exclude the possibility that some children might have had co-infections with other bacterial or viral pathogens.

## Conclusions

The present study has shown that there are no characteristic radiological findings, or routine laboratory tests that would distinguish CAP caused by MP from other CAP. Our results suggest that some clinical features are indicative for MP pneumonia, such as headache and wheezing and therefore may aid the process differentiating it from pneumonia cases caused by other pathogens. However, detection of IgM antibodies together with RT-PCR allows for precise and reliable diagnosis of MP infections in children during the acute phases of disease, indicating a possible use of both techniques as a valid diagnostic approach in early detection of MP infection in children with CAP.
